# Supplementary material for: Prognostic value of MRI volumetric parameters in non-small cell lung cancer patients after immune checkpoint inhibitor therapy: comparison with response assessment criteria
Source: Cancer Imaging. 2023 Oct 24;23:102. doi: 10.1186/s40644-023-00624-0 (PMC10594817; doi:10.1186/s40644-023-00624-0)
Supplement: Supplementary file 1 — Supplementary Material 1 [file 40644_2023_624_MOESM1_ESM.docx]

**Supplementary Table 1** MRI Parameters Used

| **Pulse Sequence** | **1.5 Tesla (n = 23)** | **3.0 Tesla (n = 40)** |
| --- | --- | --- |
| **T2WI** |  |  |
| Repetition time (ms) | 4247.18 ± 223.49 (3850–4440) | 3273.17 ± 466.53 (3000–3956) |
| Echo time (ms) | 100.36 ± 4.27 (88–103) | 80 |
| Section thickness (mm) | 5 | 5 |
| **FLAIR** |  |  |
| Repetition time (ms) | 9181.82 ± 603.02 (9000–11000) | 10142.86 ± 1069.05 (9000–11000) |
| Echo time (ms) | 114.64 ± 12.54 (109–140) | 127.93 ± 4.83 (125–135) |
| Section thickness (mm) | 5 | 5 |
| **Precontrast T1WI** |  |  |
| Repetition time (ms) | 521.2 ± 42.86 (484–598) | 446.49 ± 9.47 (425–450.2) |
| Echo time (ms) | 10.91 ± 2.02 (10–15) | 11.03 ± 0.73 (10–12) |
| Section thickness (mm) | 5 | 5 |
| **Postcontrast T1W-3D GRE** |  |  |
| Repetition time (ms) | 1680.75 ± 829.12 (8–2130) | 141.09 ± 224.69 |
| Echo time (ms) | 3.23 ± 0.28 (3–4) | 6.89 ± 4.19 |
| Section thickness (mm) | 3 | 3 |

Data are expressed as mean ± standard deviation. Numbers in parenthesis are range.

FLAIR, fluid-attenuated inversion recovery.

1.5T and 3T MRI machines were used for the baseline MRIs, and the number of patients scanned using each is included in the column headings.

**Supplementary Table 2** Summary of the mRECIST, RANO-BM, and iRANO-BM Criteria

|  | **mRECIST** | **RANO-BM** | **iRANO-BM** |
| --- | --- | --- | --- |
| **Measurable lesions** | ≥5 mm in the smallest diameter | - ≥10 mm in one diameter  - ≥5 mm in the perpendicular plane  - (≥5 mm with MRI imaging a 1.5 mm slice thickness or less) | - ≥10 mm in one diameter  - ≥5 mm in the perpendicular plane  - (≥5 mm with MRI imaging a 1.5 mm slice thickness or less) |
| **Target lesions** | 5 | 5 | 5 |
| **CR** | Complete disappearance of all lesions | - Complete disappearance of all target lesions and no new lesions  - No steroids  - Stable/improved clinical status | - Complete disappearance of all  target lesions and no new lesions  - No steroids  - Stable/improved clinical status |
| **PR** | ≥30% decrease in SLD compared to baseline | - ≥30% decrease in SLD compared to baseline and no new lesions  - Stable/decreased steroid dose  - Stable/improved clinical status | - ≥30% decrease in SLD compared  to baseline and no new lesions  - Stable/decreased steroid dose  - Stable/improved clinical status |
| **PD** | ≥20% increase in SLD compared to nadir  New lesions | - ≥20% increase in SLD compared to nadir, at least one lesion with ≥5 mm absolute increase  - New lesions*  - Worse clinical status | - ≥20% increase in SLD compared to nadir, at least one lesion with ≥5 mm absolute increase  - New lesions*  - Worse clinical status  Note:  A window of 3 months is required for patients who meet the criteria for PD within 6 months of initiating immunotherapy.  If PD are met on the follow-up scan 3 months later, the PD date is back-dated to the initial date it was identified. |
| **SD** | Does not meet criteria for CR, PR, or PD | | |

mRECIST, modified Response Evaluation Criteria in Solid Tumors; RANO-BM, Response Assessment in Neuro-Oncology Brain Metastases; iRANO-BM, immunotherapy Response Assessment in Neuro-Oncology adjusted for Brain Metastases; CR, complete response; PR, partial response; PD, progressive disease; SD, stable disease.
*For immunotherapy-based treatment, new lesions alone do not define progression. Instead, these lesions are measured and included in the sum of the longest diameters to determine a 20% increase.

**Supplementary Table 3** Statistical Power for pairwise comparisons of median PFS between criteria

| **Power** | **N** | **Alpha** | **Beta** | **Mean of Paired Differences** | **S** | **Effect Size** |
| --- | --- | --- | --- | --- | --- | --- |
| **mRECIST & RANO-BM** | | | | | | |
| 0.989 | 63 | 0.0167 | 0.011 | 2.0 | 3.39 | 0.590 |
| **mRECIST & iRANO-BM** | | | | | | |
| 0.999 | 63 | 0.0167 | 0.011 | 4.0 | 5.11 | 0.783 |
| **RANO-BM & iRANO-BM** | | | | | | |
| 0.999 | 63 | 0.0167 | 0.011 | 3.0 | 3.93 | 0.763 |

mRECIST, modified Response Evaluation Criteria in Solid Tumors; RANO-BM, Response Assessment in Neuro-Oncology Brain Metastases; iRANO-BM, immunotherapy Response Assessment in Neuro-Oncology adjusted for Brain Metastases.

Note: The null hypothesis is the mean of paired differences = 0 and the alternative hypothesis is the mean of paired differences ≠ 0. Results are from the numeric results for paired Z-tests.

**Summary Statements**

**mRECIST & RANO-BM:** A sample size of 63 achieves 99% power to detect a mean of paired differences of 2.0 with a known standard deviation of differences of 3.39 and with a significance level (alpha) of 0.0167 using a two-sided paired z-test.

**mRECIST & iRANO-BM:** A sample size of 63 achieves 99% power to detect a mean of paired differences of 4.0 with a known standard deviation of differences of 5.11 and with a significance level (alpha) of 0.0167 using a two-sided paired z-test.

**RANO-BM & iRANO-BM:** A sample size of 63 achieves 99% power to detect a mean of paired differences of 3.0 with a known standard deviation of differences of 3.93 and with a significance level (alpha) of 0.0167 using a two-sided paired z-test.

**Report Definitions**

Power is the probability of rejecting a false null hypothesis.

N is the number of pairs drawn from the population.

Alpha is the probability of rejecting a true null hypothesis.

Beta is the probability of accepting a false null hypothesis.

Mean of Paired Differences is the mean difference at which the power and sample size are determined.

S is the standard deviation of the paired differences for the population. It is assumed to be known, and the normal distribution z-test is used in place of the t-test.

Effect Size, |Mean of Paired Differences|/Sigma, is the relative magnitude of the effect under the alternative.

**References**

Machin, D., Campbell, M., Fayers, P., and Pinol, A. 1997. Sample Size Tables for Clinical Studies, 2nd Edition. Blackwell Science. Malden, MA.

Zar, Jerrold H. 1984. Biostatistical Analysis (Second Edition). Prentice-Hall. Englewood Cliffs, New Jersey.

**Supplementary Table 4** Confusion Matrices between Response Assessments

| **mRECIST** | **RANO-BM** | | | | |
| --- | --- | --- | --- | --- | --- |
|  | CR | PR | SD | PD | Total |
| CR | 2 | 0 | 3 | 0 | 5 (7.9%) |
| PR | 0 | 9 | 5 | 0 | 14 (22.2%) |
| SD | 0 | 0 | 22 | 0 | 22 (34.9%) |
| PD | 0 | 0 | 6 | 16 | 22 (34.9%) |
| Total | 2 (3.2%) | 9 (14.3%) | 36 (57.1%) | 16 (25.4%) | 63 |
| **mRECIST** | **iRANO-BM** | | | | |
|  | CR | PR | SD | PD | Total |
| CR | 2 | 0 | 3 | 0 | 5 (7.9%) |
| PR | 0 | 9 | 5 | 0 | 14 (22.2%) |
| SD | 0 | 0 | 22 | 0 | 22 (34.9%) |
| PD | 0 | 0 | 10 | 12 | 22 (34.9%) |
| Total | 2 (3.2%) | 9 (14.3%) | 40 (63.5%) | 12 (19%) | 63 |
| **RANO-BM** | **iRANO-BM** | | | | |
|  | CR | PR | SD | PD | Total |
| CR | 2 | 0 | 0 | 0 | 2 (3.2%) |
| PR | 0 | 9 | 0 | 0 | 9 (14.3%) |
| SD | 0 | 0 | 36 | 0 | 36 (57.1%) |
| PD | 0 | 0 | 4 | 12 | 16 (25.4%) |
| Total | 2 (3.2%) | 9 (14.3%) | 40 (63.5%) | 12 (19%) | 63 |

mRECIST, modified Response Evaluation Criteria in Solid Tumors; RANO-BM, Response Assessment in Neuro-Oncology Brain Metastases; iRANO-BM, immunotherapy Response Assessment in Neuro-Oncology adjusted for Brain Metastases.


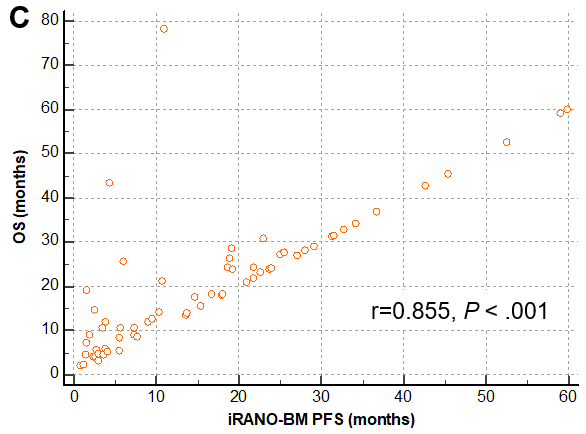

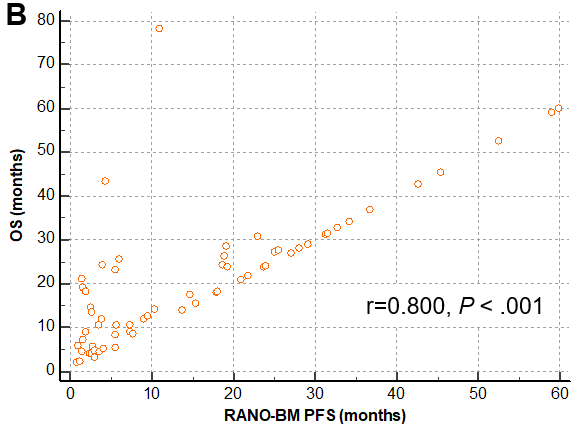
**Supplementary Figure 1** Correlation between overall survival (OS) and progression-free survival (PFS) for each classification criterion: (A) mRECIST, (B) RANO-BM, and (C) iRANO-BM. The r values indicate the Spear man's rank correlation coefficient.


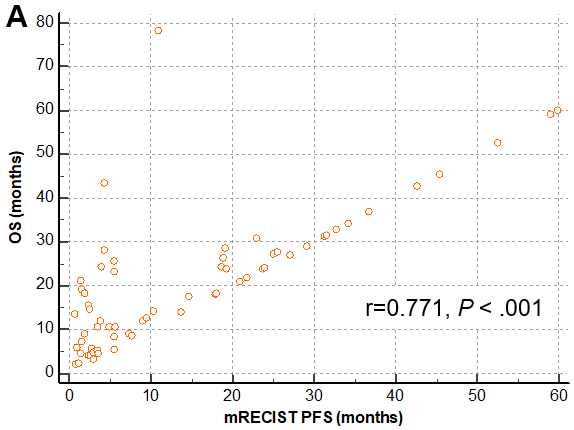


mRECIST, modified Response Evaluation Criteria in Solid Tumors; RANO-BM, Response Assessment in Neuro-Oncology Brain Metastases; iRANO-BM, immunotherapy Response Assessment in Neuro-Oncology adjusted for Brain Metastases.
