# Supplementary material for: Prognostic value of MRI volumetric parameters in non-small cell lung cancer patients after immune checkpoint inhibitor therapy: comparison with response assessment criteria
Source: Cancer Imaging. 2023 Oct 24;23:102. doi: 10.1186/s40644-023-00624-0 (PMC10594817; doi:10.1186/s40644-023-00624-0)
Supplement: Supplementary file 3 — Supplementary Material 3 [file 40644_2023_624_MOESM3_ESM.docx]

**Prognostic value of MRI volumetric parameters in non-small cell lung cancer patients after immune checkpoint inhibitor therapy: comparison with response assessment criteria**

Yun Hwa Roh^1^, Ji Eun Park^1^, Sora Kang^2^, Shinkyo Yoon^3^, Sang-We Kim^3^, Ho Sung Kim^1^

^1^Department of Radiology and Research Institute of Radiology, University of Ulsan College of Medicine, Asan Medical Center, Seoul, Republic of Korea

^2^Division of Hematology and Oncology, Department of Internal Medicine, Chungnam National University Hospital, Daejeon, Republic of Korea

^3^Department of Oncology, University of Ulsan College of Medicine, Asan Medical Center, Seoul, Republic of Korea

**Corresponding Author:**

Ji Eun Park, M.D., Ph.D.
Department of Radiology and Research Institute of Radiology, University of Ulsan College of Medicine, Asan Medical Center, 88, Olympic-ro 43-gil, Songpa-gu, Seoul 05505, Korea
Phone: 82-2-3010-1505
E-mail address: [jieunp@gmail.com](mailto:jieunp@gmail.com)

**Abstract**

**Background:** Accurate response parameters are important for patients with brain metastasis (BM) undergoing clinical trials using immunotherapy, considering poorly defined enhancement and variable responses. This study investigated MRI-based surrogate endpoints for patients with BM receiving immunotherapy.

**Methods**: Sixty-three non-small cell lung cancer patients with BM who received immune checkpoint inhibitors and underwent MRI were included. Tumor diameters were measured using a modification of the RECIST 1.1 (mRECIST), RANO-BM, and iRANO adjusted for BM (iRANO-BM). Tumor volumes were segmented on 3D contrast-enhanced T1-weighted imaging. Differences between the sum of the longest diameter (SLD) or total tumor volume at baseline and the corresponding measurement at time of the best overall response were calculated as “changes in SLDs” (for each set of criteria) and “change in volumetry,” respectively. Overall response rate (ORR), progressive disease (PD) assignment, and progression-free survival (PFS) were compared among the criteria. The prediction of overall survival (OS) was compared between diameter-based and volumetric change using Cox proportional hazards regression analysis.

**Results:** The mRECIST showed higher ORR (30.1% vs. both 17.5%) and PD assignment (34.9% vs. 25.4% [RANO-BM] and 19% [iRANO-BM]). The iRANO-BM had a longer median PFS (13.7 months) than RANO-BM (9.53 months) and mRECIST (7.73 months, *P =* .003). The change in volumetry was a significant predictor of OS (HR = 5.87, 95% CI: 1.46–23.64, *P* = 0.013). None of the changes in SLDs, as determined by RANO-BM or iRANO-BM, were significant predictors of OS, except for the mRECIST, which exhibited a weak association with OS.

**Conclusion:** Quantitative volume measurement may be an accurate surrogate endpoint for OS in patients with BM undergoing immunotherapy, especially considering the challenges of multiplicity and the heterogeneity of sub-centimeter size responses.

**Keywords:** Brain metastases, MRI, volumetric analysis, immunotherapy, non-small cell lung cancer

**Highlights:**

- The change in volumetric measurementwas a significant predictor of overall survival compared with response assessment guidelines.
- Treatment response can be objectively measured using volumetric MRI measurement of brain metastases.
- Volumetric MRI measurement is particularly useful in brain metastases, challenged by sub-centimeter size and multiplicity.

**Introduction**

Recent advances in immunotherapy, including immune checkpoint inhibitors (ICI), have improved the prognosis of patients with cancer, and patients with brain metastasis (BM) can also benefit from such treatments ^[1-3]^. A response rate of 29.7% was reported for pembrolizumab treatment of BM in patients with programmed cell death-ligand 1 (PD-L1)-positive non-small cell lung cancer (NSCLC) ^[1]^. However, correct interpretation of the response of BM following immunotherapy is challenging because the image findings can be variable, and differentiation of treatment response from tumor progression is often difficult ^[4]^. The Response Assessment in Neuro-Oncology (RANO) group has published the immunotherapy RANO (iRANO) criteria ^[5]^ to provide guidelines for immunotherapy response assessment. The essence of iRANO-based assessment is that it allows a window of 3 months before the confirmation of initial imaging suspicious for progression.

Magnetic resonance imaging (MRI)-based response criteria for clinical trials are intended to provide a standardized measurement of treatment response on MRI. In BM, response criteria typically involve assessing a 2-dimensional (2D) measurement of a lesion’s largest diameter on MRI, with such criteria including the Response Evaluation Criteria in Solid Tumors 1.1 (RECIST 1.1) and the Response Assessment in Neuro-Oncology Brain Metastases (RANO-BM) ^[6-9]^. Both RECIST 1.1 and RANO-BM require a measurable lesion to be at least 10 mm in diameter. However, in clinical practice, many BMs are less than 10 mm ^[7]^, and the tumor burden from such lesions can be important when they are numerous. A recent study on brain glioma ^[10]^ showed that volumetric measurement of tumor burden enabled objective assessment of tumor response. Volumetric measurement of BMs, regardless of their size and numbers, may provide a surrogate endpoint for a patient’s outcome, but few studies have applied such quantification or compared results with other response criteria in patients undergoing immunotherapy.

We hypothesized that volumetric analysis on MRI would provide an accurate surrogate endpoint for response assessment in patients with BM, especially in patients with NSCLC and BM treated with ICI. There is no iRANO-based assessment for BM, thus we tried to apply the iRANO criteria to BM using the sum of the longest diameters (iRANO adjusted for BM, iRANO-BM in this manuscript). Among different response criteria for BM, we first compared the central nervous system (CNS) response assessment of a modified RECIST (mRECIST) ^[11]^ and RANO-BM and iRANO-BM on central review and then evaluated the predictive value of the response criteria and volumetric measurements for patient survival. The purpose of this study was to investigate an accurate MRI-based surrogate endpoint for response assessment in patients with BM who have undergone immunotherapy.

**Materials and Methods**

*Patients*

This retrospective study was approved by the institutional review board of Asan Medical Center, and the requirement for informed consent was waived (IRB No.2021-1300). We identified patients with NSCLC who underwent ICI monotherapy at Asan Medical Center between January 2014 and April 2021 and who had BM at the time of initiation of index ICI therapy. Between January 2014 and April 2021, 87 patients with NSCLC and brain metastasis were treated with ICI monotherapy. From these, patients were excluded because of the following: received immunotherapy before the administration of index ICI (n = 4); received only one dose of ICI because of death (n = 1), pneumonia (n = 3), or disease progression before the administration of initial ICI (n = 2); lung cancer with synchronous breast cancer (n = 1); bone metastasis (n = 1); baseline MRI was not available prior to the administration of initial ICI (n = 2); and absence of adequate follow-up MRI for response evaluation (n = 10). Finally, a total of 63 patients (median age, 63 years; range, 42–80; 12 female) were included in the study.

*Imaging Acquisition*

Brain MRI was obtained with either 1.5 or 3 T scanners and included T2-weighted, T2-weighted FLAIR, and precontrast and postcontrast T1-weighted images. The imaging parameters are summarized in **Supplementary Table 1**. An MRI within 2 months of ICI initiation was considered the baseline brain imaging, and follow-up MRIs were obtained every 4–8 weeks after initiating therapy.

*Study Design*

The study had two co-primary purposes. The first purpose was to measure and compare responses using different response criteria: RECIST, RANO-BM, and iRANO-BM for each patient. The second purpose was to investigate whether there is an association between the initial response measured by the subtraction method of the sum of longest diameter (SLD)—indicated by the change in the diameter of target lesions between the BOR date and the baseline date—and overall survival.

*Response Assessment Using Different Response Criteria*

Image analysis was performed by two neuroradiologists acting as central readers (J.E.P. and H.S.K., with 9 and 24 years of experience in neuro-oncologic imaging, respectively), who were blinded to the outcomes of the patients. The readers measured the tumors on baseline MRI and follow-up MRI and determined the patients’ responses by comparing the baseline and follow-up images. The date of best overall response (BOR), which was defined as the single best response status across all response evaluation time points until disease progression, and date of progression were recorded by the readers.

The readers chose five target measurable lesions for mRECIST (≥ 5 mm in the smallest diameter) and RANO-BM/iRANO-BM (≥ 10 mm in one diameter) assessments. If there were more than five lesions present, the target lesions were chosen in descending order of size; iRANO adjusted for BM (iRANO-BM) was applied by measuring the longest diameter. Any discrepancies in the choice of target lesions were solved through discussion. The locations of the target lesions were recorded, and their diameters were measured on MRI. The SLDs of the five target lesions was calculated for mRECIST, RANO-BM, and iRANO-BM. Notably, RANO-BM allows the use of a 5 mm cutoff if the slice thickness is equal to or less than 1.5 mm. In this study, the slice thicknesses were 3 mm, and the 10 mm cutoff was adopted.

**Supplementary Table 2** summarizes the response assessment criteria of mRECIST, RANO-BM, and iRANO-BM for complete response (CR), partial response (PR), stable disease (SD), and progressive disease (PD). The overall response rate (ORR) was defined as CR plus PR rates, and the disease control rate was defined as CR plus PR plus SD rates. **Figure 1** shows representative cases showing different response assessments according to each set of response assessment criteria.

*Quantitative Assessment: Changes in SLDs and Volumetry*

For the mRECIST, RANO-BM, and iRANO-BM criteria, the “changes in SLDs” were calculated as follows: (*SLD of the target lesions at the BOR date*) − (*SLD of the target lesions at the baseline date*). A similar calculation was applied for volumetric measurement: (*total tumor volume at BOR date*) − (*total tumor volume at baseline date*). The BOR date of the volumetric assessment was taken to be the time point of the smallest sum of the lesion volumes.

There was a 3-month wash-out period between response assessment and volumetric measurement. All images were anonymized, and the readers were blind to any clinical information. For volumetric analysis, the MRI examinations were subjected to preprocessing including resampling to 1 × 1 × 1 mm and co-registration performed using AFNI software (Analysis of Functional NeuroImages) ^[12]^. Segmentation of the enhancing tumor region was performed by a neuroradiologist (Y.H.R., with 1 year of experience in neuro-oncological imaging) using a semi-automatic procedure on the 3D contrast-enhanced T1-weighted imaging. This procedure used a segmentation threshold and a region-growing segmentation algorithm implemented using MITK software ([*www*.*mitk*.*org*](http://www.mitk.org) German Cancer Research Center, Heidelberg, Germany) ^[13]^. For volumetric analysis, all measurable and nonmeasurable enhancing lesions were segmented and summed. All segmented images were validated by an experienced neuroradiologist (J.E.P.) after central reading.

Since patients identified with PD at the initial follow-up MRI did not continue ICI after only one course of ICI therapy, these patients were excluded (n = 6) from the OS analysis because of the short follow-up, limited effect of ICI therapy on OS, and confounding factors of other treatments initiated after ICI termination.

*Outcomes*

The primary endpoints were the associations between OS and the changes in SLDs, as well as change in volumetry. Overall survival was calculated from the date of baseline imaging to the date of death from any cause. The date of death was obtained from the national healthcare data linked to our hospital, and the date of the last follow-up MRI was considered the censored date.

For the secondary endpoints, we compared the median progression-free survival (PFS), ORR, and PD assignments according to the mRECIST, RANO-BM, and iRANO-BM criteria. Progression-free survival was calculated as the period from the date of the baseline imaging to the date of progression or death due to any cause, or if no progression was observed, from baseline imaging date to the censored date.

*Statistical Analysis*

*Comparisons between response criteria:* To compare the level of agreement, a weighted kappa analysis was performed using Fleiss-Cohen quadratic weights. The agreement between two response criteria was categorized as follows: poor (κ = 0–0.20), mild (κ = 0.21–0.40), moderate (κ = 0.41–0.60), substantial (κ = 0.61–0.80), and almost perfect (κ > 0.80).

The proportions of the best CNS responses according to mRECIST, RANO-BM, and iRANO-BM classifications were analyzed. The McNemar test was used to compare differences in the proportions of patients defined with PD according to each set of criteria.

*Comparison of clinical outcomes:* The OS and PFS results are reported as medians with 95% confidence intervals (CIs). Differences in PFS between the criteria were calculated using the Wilcoxon signed-rank test, and the results were corrected for multiple comparisons using the Bonferroni adjustment (resulting in a *P*-value of < 0.017 being considered significant).

Statistical power for the pairwise comparisons of median PFS between criteria was estimated using paired z-tests (PASS 15.0.7. version). For example, between mRECIST and RANO-BM, with a sample size of 63, an assumed mean of paired differences of 2 months, and a calculated standard deviation of 3.39 months, a statistical power of 99% was achieved to detect this mean difference in paired differences. The statistical power calculations are summarized in **Supplementary Table 3.**

Survival curves for OS were drawn for each set of response criteria using the Kaplan-Meier method. A log-rank test was used to determine differences in OS between PD and non-PD patients as defined by each set of response criteria.

The correlation between OS and PFS for each criterion was evaluated using Spearman's rank correlation coefficient.

*Prediction of OS using quantitative measurements:* Cox proportional hazards regression analysis was applied to the changes in SLDs and change in volumetry as a single covariable to determine the association with OS. Hazard ratios (HRs) and their corresponding 95% CIs were also calculated.

Descriptive statistics were used to analyze categorical and continuous variables of the patient demographics. For continuous variables, the normality of the distribution was assessed using the Kolmogorov-Smirnov test. Normally distributed numerical variables are presented as mean and standard deviation, and non-normally distributed numerical variables are presented as median and range.

For all statistical analyses except those that were Bonferroni corrected, two-sided *P*-values < 0.05 were considered statistically significant. All statistical analysis was conducted using R statistical software (version 4.1.3, Vienna, Austria) and Medcalc software (version 20.115, Ostend, Belgium).

**Results**

*Baseline Characteristics*

The baseline characteristics of the study population are described in **Table 1**. Among the 63 included patients (median age: 63 years; age range: 42–80; 12 female), 47 patients (74.6%) were stage IV and 16 patients (25.4%) were stage I–III at the time of the initial diagnosis of NSCLC. Histology revealed adenocarcinoma to be the most common type (44 patients, 69.8%), followed by squamous cell carcinoma (14 patients, 22.2%). The remaining five patients were confirmed as giant cell carcinoma, adenosquamous cell carcinoma, sarcomatoid carcinoma, or large cell neuroendocrine carcinoma.

Local brain treatment before receiving ICI included tumor resection, local radiotherapy, and whole brain radiotherapy performed in 7 (11.1%), 44 (69.8%), and 4 (6.3%) patients, respectively. Sixteen patients (25.4%) did not receive any local CNS treatment before starting ICI.

Among the 63 patients, 27 (42.9%) received pembrolizumab, 18 (28.6%) received nivolumab, and 18 (28.6%) received atezolizumab. Additionally, 15 patients were administered steroids during ICI treatment. During the immunotherapy, 20 patients (31.7%) underwent concomitant local radiotherapy, and 1 received whole-brain radiotherapy. The remaining 42 patients (66.7%) did not receive local treatment. Patients who were treated with SRS had a follow-up period of more than 6 months, and no instances of radiation necrosis were observed during the course of ICI treatment. The time from diagnosis to initiation of immune checkpoint inhibitor therapy was 20.7 ± 34.14 (mean ± standard deviation) months.

*Comparisons Between mRECIST, RANO-BM, and iRANO-BM Criteria*

The mRECIST showed moderate to substantial agreement with RANO-BM and iRANO-BM (κ = 0.69 and 0.57, respectively), and there was almost perfect agreement between RANO-BM and iRANO-BM (κ = 0.87). Comparisons of the CNS response according to each set of criteria are shown in **Table 2** and **Figure 2**. Confusion matrices for each pairing of response assessments are shown in **Supplementary Table 4**. The ORR was higher with mRECIST (30.1%) than with RANO-BM (17.5%) or iRANO-BM (17.5%), but the disease control rate was lower with mRECIST than with RANO-BM and iRANO-BM (65% vs. 74.7% vs. 81%, respectively).

PD was more frequently assigned using mRECIST (34.9%, 22/63) than using RANO-BM (25.4%, 16/63) or iRANO-BM (19%, 12/63). The differences between mRECIST and RANO-BM, as well as between mRECIST and iRANO-BM, were statistically significant (*P* = 0.031 and *P* = 0.002, respectively). However, there was no significant difference between RANO-BM and iRANO-BM (*P* = 0.125).

*Comparison of Clinical Outcomes Between the Response Criteria*

**Table 3** shows the differences in median PFS when PFS was defined according to the different response criteria. The median PFS values as determined by mRECIST, RANO-BM, and iRANO-BM were 7.73 months (95% CI, 4.52–18.05 months), 9.53 months (95% CI, 5.53–18.55 months), and 13.7 months (95% CI, 7.49–19.07 months), respectively. There was no significant difference in the determined PFS between mRECIST and RANO-BM or between RANO-BM and iRANO-BM. However, the PFS as determined by iRANO-BM was significantly longer than that determined by mRECIST (*P* = 0.001). A power analysis indicated sufficient statistical power for the pairwise comparisons between criteria **(Supplementary Table 3**).

The median OS of the study population was 23.7 months (95% CI, 14.6–28.5 months). **Figure 3** shows Kaplan-Meier survival curves for the patients for each response assessment criteria; there was a significant difference in OS between PD and non-PD groups according to all three response criteria (log-rank test, largest *P* < 0.05).

The relationship between OS and PFS for each response criterion is illustrated in **Supplementary Figure 1.** PFS exhibited a strong correlation with OS using all criteria—mRECIST (*r*_s_ = 0.771, 95% CI 0.647–0.855, *P* < 0.001), RANO-BM (*r*_s_ = 0.800, 95% CI 0.689–0.875, *P* < 0.001), and iRANO-BM (*r*_s_ = 0.855, 95% CI 0.770–0.910, *P* < 0.001)—based on Spearman’s rank correlation coefficient.

*Prediction of OS Using Quantitative Measurement*

Associations between OS and changes in SLDs and change in volumetry are shown in **Table 4**. Changes in SLDs according to RANO-BM (HR 1.07, 95% CI: 0.99–1.16, *P* = 0.077) or iRANO-BM criteria (HR 1.03, 95% CI: 0.99–1.07, *P* = 0.172) did not demonstrate a predictive value for OS, except for the mRECIST exhibited a weak association for OS (HR 1.07, 95% CI: 1.005–1.14, *P* = 0.035). On the other hand, the change in volumetry was a significant predictor of OS, with a quantitative total volume increase indicating shorter OS (HR 5.87, 95% CI: 1.46–23.64, *P* = 0.013). **Figure 4** demonstrates cases for which the 3D response was useful for predicting OS.

**Discussion**

With improved survival of patients with BM, reliable response parameters for differentiating patients according to treatment response and tumor progression are very important for clinical trials and clinical use. In this study, we evaluated different CNS response assessment criteria and compared their clinical outcomes in patients with NSCLC who were treated for BM with ICI. Also, we measured quantitative changes in SLDs and volumetry and analyzed their significance to predict OS. The mRECIST criteria showed a higher ORR and PD assignment rate than RANO-BM or iRANO-BM. Median PFS was longest according to iRANO-BM, followed by RANO-BM and then mRECIST. For quantitative assessment, we found that change in volumetry, which refers to the total tumor volume decrease after ICI, was a significant predictor of OS, suggesting the possible role of volumetric assessment as a surrogate endpoint in patients with BM.

Although 2D measurements of the longest diameter are simple to make and are readily utilized in clinical practice, previous studies demonstrated volumetric measurements to be more reliable and accurate than 2D-based approaches ^[10,14,15]^. In this study, we calculated changes in SLD (2D) and volumetry (3D), which can be considered initial tumor responses to treatment, and found that change in volumetry was a significant predictor of OS, whereas 2D-based measurement was not. This discrepancy can be attributed to two primary factors. First, volumetric measurements may offer a more accurate representation of changes in tumor burden than 2D diameter changes. Second, volumetric assessments included all lesions, whereas 2D-based measurements were restricted to target lesions. In routine clinical practice, it is often impractical to measure all lesions, especially those with sub-millimeter diameters, and consistent tracking of these small lesions in follow-up imaging presents additional challenges. Given these considerations, volumetric analysis stands out as a valuable method for the more accurate and straightforward assessment of lesions.

Previous studies report inconsistent results for associations between volumetric methods and clinical outcomes in glioma. Volumetric methods did not provide improved prediction of OS compared with the 2D-RANO criteria in the first 12 weeks of bevacizumab treatment in patients with recurrent glioblastoma ^[16]^, and volumetric methods exhibited similar PFS to modified RANO criteria in patients with recurrent glioblastoma treated with immunotherapy ^[17]^. By contrast, a large retrospective cohort study of patients with glioma ^[10]^ showed that automated volumetric tumor measurement was superior to 2D RANO measurement for predicting OS.

The results of our study are specific to BM, which has different properties to other brain tumors. The majority of patients with BM are found to have multiple rather than single lesions on presentation, with many lesions of small diameter ^[18]^, making volumetric assessment more feasible for reflecting the total tumor burden. Furthermore, a dissociated response with the coexistence of responding and nonresponding lesions was reported for 3.3–9.2% of patients treated with ICIs ^[19,20]^. This may be due to genomic tumor heterogeneity and differences in the tumor microenvironment ^[21]^. In cases of multiple BM lesions showing variable size changes on imaging, volumetric assessment may provide a useful approach allowing changes in the total tumor volume to be tracked. We adopted a subtraction-based method and not a ratio-based method ([*diameters at BOR* − *diameters at baseline*]/*diameter at baseline*) because in patients with BM, multiple small lesions are present at baseline and these are considered non-measurable under the RANO-BM criteria, which yields a denominator of 0. For volumetric analysis, both the subtraction-based method and the ratio (percentage)-based method are feasible.

Few studies have compared the different response assessment criteria in patients with BM who underwent immunotherapy. One major difference between the mRECIST, RANO-BM, and iRANO-BM criteria is the size limit for measurable lesions. The mRECIST criteria define the limit of a measurable lesion as 5 mm or more, while RANO-BM defines the minimum size as 10 mm. Qian et al. ^[11]^ compared mRECIST, RECIST 1.1, RANO-BM, and RANO-HGG in 36 patients with BM enrolled in an ongoing trial of pembrolizumab. In their study, 13 patients with no lesions ≥10 mm in diameter were excluded because the target lesion was ineligible according to RANO-BM. After excluding these patients, no difference was seen between the different criteria for categorizing PD. In our study, the sizes of the target lesions were between 5 and 10 mm in 11.1% (7/63) of patients, resulting in different response assessments between mRECIST and RANO-BM or iRANO-BM.

A second major difference between the response criteria is the definition of disease progression. While mRECIST defines new lesions as PD, RANO-BM requires measurement of such lesions and inclusion in the SLD used to determine PD in patients receiving immunotherapy. The iRANO criteria differ from those of RANO-BM in that they are developed for glioma and state that progression is differentiated from pseudoprogression via repeated imaging after 3 months if PD is suspected within 6 months of ICI initiation. In our study, more patients were assessed as PD with mRECIST (22/63) than with RANO-BM (16/63) or iRANO-BM (12/63). There were six patients assigned as having PD by mRECIST but not by RANO-BM. Of these patients, RANO-BM classified five as exhibiting SD and one as having a PR. Among these six patients, one had a PD including non-target lesions according to RANO-BM. The median PFS was significantly longer with iRANO-BM than with mRECIST (13.7 months vs. 7.73 months, *P* = 0.001). Of the 15 patients who showed PD according to RANO-BM and iRANO-BM in the first follow-up scan within 6 months of ICI initiation, five were later confirmed as SD by iRANO-BM at the subsequent follow-up imaging, whereas the rest of the patients had delayed confirmation of PD. In accordance with previous studies ^[17,22]^, our results demonstrated no significant difference in the determination of PD and median PFS between RANO-BM and iRANO-BM. All three response assessment criteria examined in our study showed significant differences in OS between PD and non-PD patients, but no method was superior to the others at predicting OS.

Our study has several limitations. First, it is a retrospective study performed in a single center with the MRI scanners and acquisition parameters used differing across the patients. Second, only MRI measurement was used for response assessment, with the clinical status and steroid dosage not being considered because the main purpose of our study was to evaluate the use of imaging parameters for predicting survival outcomes. Third, the volumetric segmentation was performed semiautomatically; currently, deep learning-based algorithms enabling fully automated detection and quantification of tumor burden are being developed ^[10,23]^, and future studies should evaluate them in terms of tumor response and clinical decision-making. Despite its limitations, our study has strength in that we compared different CNS response criteria, including volumetric analysis, in a homogeneous population of patients with NSCLC and BM who received immunotherapy.

**Conclusion**

In conclusion, quantitative volumetric measurement of BM may become an accurate surrogate endpoint for OS in patients with BM undergoing immunotherapy. Our findings suggest that the response of BM to treatment can be objectively measured in clinical trials of immunotherapy, accommodating the challenges of multiplicity and the heterogeneity of sub-centimeter size responses.

**Availability of data and materials**

The dataset used or analyzed during the current study are available from the corresponding author on reasonable request.

**Abbreviations**

ICI = immune checkpoint inhibitors

BM = brain metastasis

PD-L1 = programmed cell death-ligand 1

NSCLC = non-small cell lung cancer

RANO = Response Assessment in Neuro-Oncology

iRANO = Immunotherapy Response Assessment in Neuro-Oncology

MRI = magnetic resonance imaging

2D = 2-dimenional

mRECIST = modified Response Evaluation Criteria in Solid Tumors

RANO-BM = Response Assessment in Neuro-Oncology Brain Metastases

iRANO-BM = iRANO adjusted for BM

CR = complete response

PR = partial response

SD = stable disease

PD = progressive disease

ORR = overall response rate

SLD = sum of the longest diameter

BOR = best overall response

OS = overall survival

PFS = progression-free survival

**Ethical approval and consent to participate:** This study was performed in line with the principles of the Declaration of Helsinki. Approval was granted by the institutional review board of Asan Medical Center (IRB No.2021-1300).

**Consent to publication:** Informed consent was waived by the ethics committee (IRB No.2021-1300).

**Availability of data and materials:** The datasets generated during and/or analyzed during the current study are available from the corresponding author on reasonable request.

**Competing interests:** The authors do not have any competing interest.

**Funding:** This research was supported by a grant from the Ministry of Health & Welfare, Republic of Korea (grant number: HI21C1161 and HI22C0471) and Biomedical Research Institute grant, Kyungpook National University Hospital (2015).

**Authors’ contributions:** All authors read and approved the final manuscript. Y.H.R.: Manuscript writing, data provision. J.E.P.: Manuscript editing, supervised image segmentation. S.K.: Database construction S.Y.: Database construction and data provision. S.W.K.: Clinical oversight. H.S.K.: Conceptual feedback.

**Acknowledgements:** None.

**References**

[1] Goldberg SB, Schalper KA, Gettinger SN, Mahajan A, Herbst RS, Chiang AC, et al. Pembrolizumab for management of patients with NSCLC and brain metastases: long-term results and biomarker analysis from a non-randomised, open-label, phase 2 trial. Lancet Oncol. 2020; 21: 655. PMid:32251621.

[2] Tawbi HA, Forsyth PA, Algazi A, Hamid O, Hodi FS, Moschos SJ, et al. Combined Nivolumab and Ipilimumab in Melanoma Metastatic to the Brain. N Engl J Med. 2018; 379: 722. PMid:30134131.

[3] Wu YL, Ahn MJ, Garassino MC, Han JY, Katakami N, Kim HR, et al. CNS Efficacy of Osimertinib in Patients With T790M-Positive Advanced Non-Small-Cell Lung Cancer: Data From a Randomized Phase III Trial (AURA3). J Clin Oncol. 2018; 36: 2702. PMid:30059262.

[4] Galldiks N, Kocher M, Ceccon G, Werner JM, Brunn A, Deckert M, et al. Imaging challenges of immunotherapy and targeted therapy in patients with brain metastases: response, progression, and pseudoprogression. Neuro Oncol. 2020; 22: 17. PMid:31437274.

[5] Okada H, Weller M, Huang R, Finocchiaro G, Gilbert MR, Wick W, et al. Immunotherapy response assessment in neuro-oncology: a report of the RANO working group. Lancet Oncol. 2015; 16: e534. PMid:26545842.

[6] Eisenhauer EA, Therasse P, Bogaerts J, Schwartz LH, Sargent D, Ford R, et al. New response evaluation criteria in solid tumours: Revised RECIST guideline (version 1.1). European Journal of Cancer. 2009; 45: 228. PMid:WOS:000262948300002.

[7] Lin NU, Lee EQ, Aoyama H, Barani IJ, Barboriak DP, Baumert BG, et al. Response assessment criteria for brain metastases: proposal from the RANO group. Lancet Oncol. 2015; 16: e270. PMid:26065612.

[8] Miller AB, Hoogstraten B, Staquet M, Winkler A. Reporting results of cancer treatment. Cancer. 1981; 47: 207. PMid:7459811.

[9] Macdonald DR, Cascino TL, Schold SC, Jr., Cairncross JG. Response criteria for phase II studies of supratentorial malignant glioma. J Clin Oncol. 1990; 8: 1277. PMid:2358840.

[10] Kickingereder P, Isensee F, Tursunova I, Petersen J, Neuberger U, Bonekamp D, et al. Automated quantitative tumour response assessment of MRI in neuro-oncology with artificial neural networks: a multicentre, retrospective study. Lancet Oncol. 2019; 20: 728. PMid:30952559.

[11] Qian JM, Mahajan A, Yu JB, Tsiouris AJ, Goldberg SB, Kluger HM, et al. Comparing available criteria for measuring brain metastasis response to immunotherapy. J Neurooncol. 2017; 132: 479. PMid:28275886.

[12] Cox RW. AFNI: software for analysis and visualization of functional magnetic resonance neuroimages. Comput Biomed Res. 1996; 29: 162. PMid:8812068.

[13] Nolden M, Zelzer S, Seitel A, Wald D, Muller M, Franz AM, et al. The Medical Imaging Interaction Toolkit: challenges and advances : 10 years of open-source development. Int J Comput Assist Radiol Surg. 2013; 8: 607. PMid:23588509.

[14] Chow DS, Qi J, Guo X, Miloushev VZ, Iwamoto FM, Bruce JN, et al. Semiautomated volumetric measurement on postcontrast MR imaging for analysis of recurrent and residual disease in glioblastoma multiforme. AJNR Am J Neuroradiol. 2014; 35: 498. PMid:23988756.

[15] Sorensen AG, Patel S, Harmath C, Bridges S, Synnott J, Sievers A, et al. Comparison of diameter and perimeter methods for tumor volume calculation. J Clin Oncol. 2001; 19: 551. PMid:11208850.

[16] Gahrmann R, van den Bent M, van der Holt B, Vernhout RM, Taal W, Vos M, et al. Comparison of 2D (RANO) and volumetric methods for assessment of recurrent glioblastoma treated with bevacizumab-a report from the BELOB trial. Neuro Oncol. 2017; 19: 853. PMid:28204639.

[17] Heugenhauser J, Galijasevic M, Mangesius S, Goebel G, Buchroithner J, Erhart F, et al. MRI Response Assessment in Glioblastoma Patients Treated with Dendritic-Cell-Based Immunotherapy. Cancers (Basel). 2022; 14. PMid:35326730.

[18] Suh JH. Stereotactic radiosurgery for the management of brain metastases. N Engl J Med. 2010; 362: 1119. PMid:20335588.

[19] Berz AM, Dromain C, Vietti-Violi N, Boughdad S, Duran R. Tumor response assessment on imaging following immunotherapy. Front Oncol. 2022; 12: 982983. PMid:36387133.

[20] Borcoman E, Kanjanapan Y, Champiat S, Kato S, Servois V, Kurzrock R, et al. Novel patterns of response under immunotherapy. Ann Oncol. 2019; 30: 385. PMid:30657859.

[21] Humbert O, Chardin D. Dissociated Response in Metastatic Cancer: An Atypical Pattern Brought Into the Spotlight With Immunotherapy. Frontiers in Oncology. 2020; 10. PMid:WOS:000576474200001.

[22] Chen X, Lim-Fat MJ, Qin L, Li A, Bryant A, Bay CP, et al. A Comparative Retrospective Study of Immunotherapy RANO Versus Standard RANO Criteria in Glioblastoma Patients Receiving Immune Checkpoint Inhibitor Therapy. Front Oncol. 2021; 11: 679331. PMid:34249718.

[23] Pfluger I, Wald T, Isensee F, Schell M, Meredig H, Schlamp K, et al. Automated detection and quantification of brain metastases on clinical MRI data using artificial neural networks. Neurooncol Adv. 2022; 4: vdac138. PMid:36105388.

**Table 1.** Baseline Clinical Characteristics of the Study Population

| **Characteristics** | **(n = 63)** |
| --- | --- |
| Age, median (range) | 63 (42–80) |
| Sex (male: female) | 51:12 |
| Smoking history |  |
| Never smoker | 19 (30.2%) |
| Past smoker | 22 (34.9%) |
| Current smoker | 22 (34.9%) |
| Stage at initial diagnosis of NSCLC |  |
| I–III | 16 (25.4%) |
| IV | 47 (74.6%) |
| Time between diagnosis and initiation of ICI (months, mean ± standard deviation) | 20.7 ± 34.14 |
| Histology |  |
| Adenocarcinoma | 44 (69.8%) |
| Squamous cell carcinoma | 14 (22.2%) |
| Others* | 5 (7.9%) |
| Previous CNS treatment** |  |
| None | 16 (25.4%) |
| Surgery | 7 (11.1%) |
| Local radiotherapy (GKRS or CKRS) | 44 (69.8%) |
| WBRT (≥3 months prior to immunotherapy) | 4 (6.3%) |
| CNS local treatment during immunotherapy |  |
| None | 42 (66.7%) |
| Surgery | 0 (0%) |
| Local radiotherapy (GKRS or CKRS) | 20 (31.7%) |
| WBRT | 1 (1.6%) |
| Type of immunotherapy |  |
| Pembrolizumab | 27 (42.9%) |
| Nivolumab | 18 (28.6%) |
| Atezolizumab | 18 (28.6%) |

* Includes giant cell carcinoma (n = 1), adenosquamous cell carcinoma (n = 1), sarcomatoid carcinoma (n = 2), and large cell neuroendocrine carcinoma (n = 1). ** Some patients underwent more than one CNS treatment modality.

NSCLC, non-small cell lung cancer; CNS, central nervous system; GKRS, gamma knife stereotactic radiosurgery; CKRS, cyberknife stereotactic radiosurgery; WBRT, whole brain radiotherapy.

**Table 2.** Best CNS Response According to mRECIST, RANO-BM, and iRANO-BM (n=63)

|  | **mRECIST** | **RANO-BM** | **iRANO-BM** |
| --- | --- | --- | --- |
| **Overall response rate** | 30.1% (19) | 17.5% (11) | 17.5% (11) |
| **Disease control rate** | 65% (41) | 74.7% (47) | 81% (51) |
| **CR** | 7.9% (5) | 3.2% (2) | 3.2% (2) |
| **PR** | 22.2% (14) | 14.3% (9) | 14.3% (9) |
| **SD** | 34.9% (22) | 57.1% (36) | 63.5% (40) |
| **PD** | 34.9% (22) | 25.4% (16) | 19% (12) |

mRECIST, modified Response Evaluation Criteria in Solid Tumors; RANO-BM, Response Assessment in Neuro-Oncology for Brain Metastases; iRANO-BM, immunotherapy Response Assessment in Neuro-Oncology adjusted for Brain Metastases; CR, complete response; PR, partial response; SD, stable disease; PD, progressive disease.

**Table 3.** Difference in Progression-Free Survival (PFS) according to Response Criteria

| Response Criteria | Median PFS, Months | 95% CI | *P*-value for PFS Differences | | |
| --- | --- | --- | --- | --- | --- |
|  |  |  | mRECIST | RANO-BM | iRANO-BM |
| mRECIST | 7.73 | 4.52–18.05 | - | 0.031 | **0.001** |
| RANO-BM | 9.53 | 5.53–18.55 | 0.031 | - | 0.031 |
| iRANO-BM | 13.7 | 7.49–19.07 | **0.001** | 0.031 | **-** |

Calculated *P*-values (Wilcoxon signed rank test) for differences in PFS between assessment criteria are corrected for multiple comparisons (Bonferroni’s adjustment), and a *P*-value <0.017 is considered significant. PFS, progression-free survival; CI, confidence interval.

**Table 4.** Prediction of Overall Survival According to the Difference in Quantitative Measurements between Best Overall Response (BOR) and Baseline MRI

| Quantitative Assessment | Overall Survival | | |
| --- | --- | --- | --- |
| **Changes in sum of the longest diameters (SLD) (mm)** | Hazard Ratio | 95% CI | *P*-value |
| mRECIST | 1.07 | 1.005–1.14 | **0.035** |
| RANO-BM | 1.07 | 0.99–1.16 | 0.077 |
| iRANO-BM | 1.03 | 0.99–1.07 | 0.172 |
| **Change in volumetry (mm^3^)** |  |  |  |
| Total volume of BM | 5.87 | 1.46–23.64 | **0.013** |

Note: Changes in sum of the longest diameters (SLDs) indicates the value of the baseline sum of the longest diameters (SLD) minus the SLD at the date of the BOR, and change in volumetry indicates the total volume at baseline minus the total volume at BOR date.

Hazard ratios reported here indicate the relative change in hazard that a 1 unit (10 000 voxels) increase in each imaging parameter incurs. BM, brain metastases.

**Figures and Figure legends**

**
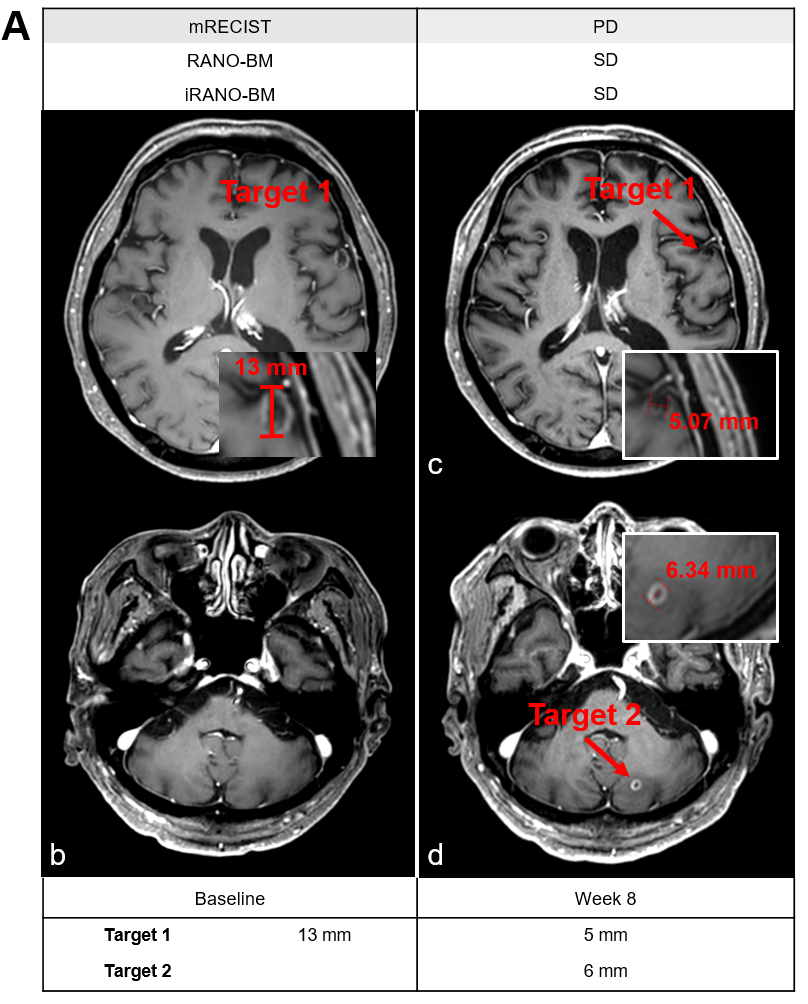
**

**Figure 1.** Baseline and Follow-up MRI Scans of a Patient with Different Response Assessments

**A.** Baseline scan (a, b) shows a target lesion of 13 mm. First follow-up scan at week 8 (c, d) shows that the target lesion has decreased in size from 13 mm to 5 mm, and that a new lesion of 6 mm has appeared. Since a new lesion has developed, progressive disease (PD) is defined by mRECIST. However, according to the RANO-BM and iRANO-BM criteria, the diameter of the new lesion is added to the sum of the longest diameter (SLD), which makes the SLD 11 mm for the follow-up scan. Therefore, the response assessment would be stable disease (SD) by RANO-BM and iRANO-BM.


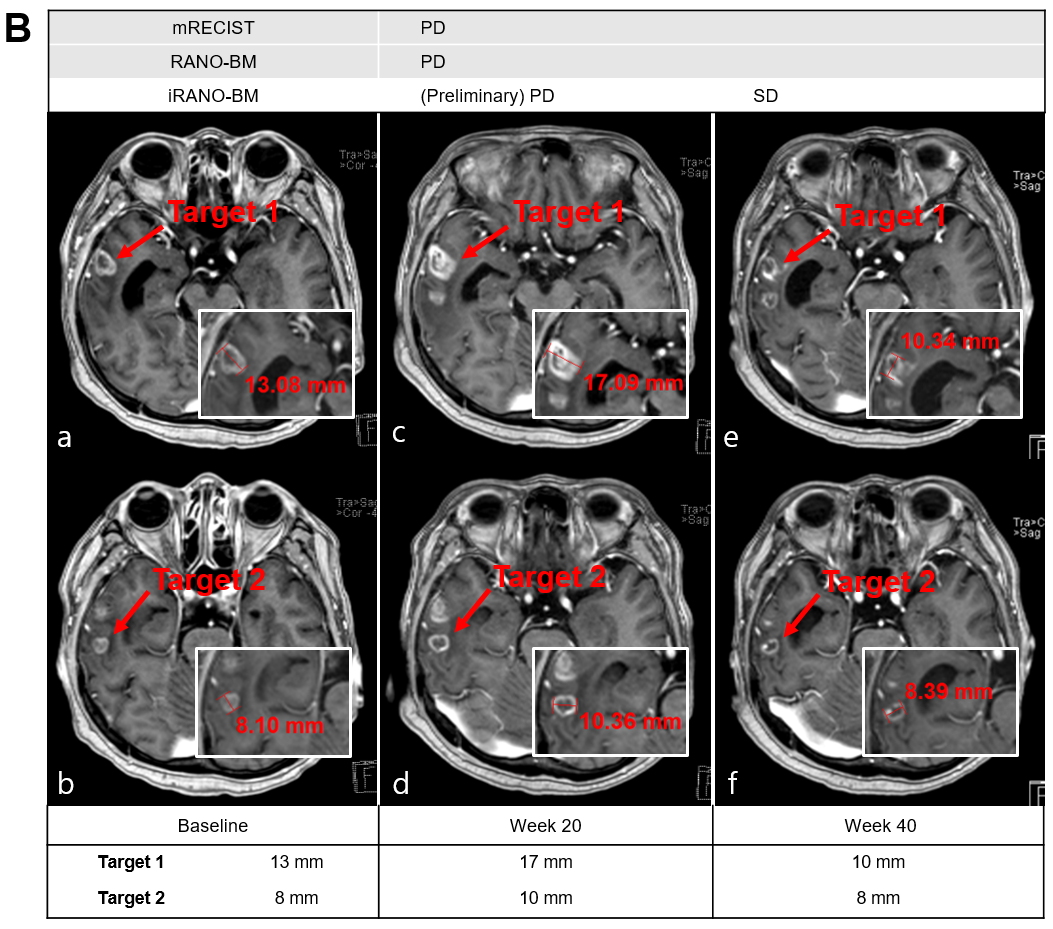


**B.** Baseline scan (a, b) shows two target lesions with sizes of 13 and 8 mm. On the first follow-up scan at week 20 (c, d), the size of each target lesion has increased, and the response assessment would be progressive disease (PD) by mRECIST and RANO-BM. However, this MRI was taken within 6 months of immunotherapy-treatment initiation, and follow-up imaging after 3 months is required for iRANO-BM assessment. In the follow-up MRI at week 40 (e, f), the size of the target lesions has decreased, and this patient is diagnosed with stable disease (SD) by iRANO-BM.


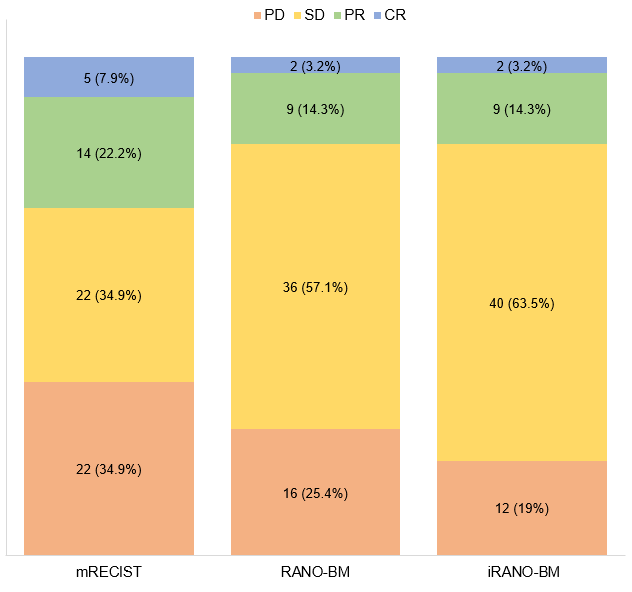


**Figure 2.** Comparison of mRECIST, RANO-BM, and iRANO-BM Criteria.

mRECIST, modified Response Evaluation Criteria in Solid Tumors; RANO-BM, Response Assessment in Neuro-Oncology Brain Metastases; iRANO-BM, immunotherapy Response Assessment in Neuro-Oncology adjusted for Brain Metastases.

**Figure 3.** Kaplan-Meier Curves of Patients with Progressive Disease (PD) Versus Nonprogressive Disease (non-PD) for Each of the Classification Criteria (A) mRECIST, (B), RANO-BM, and (C) iRANO-BM.


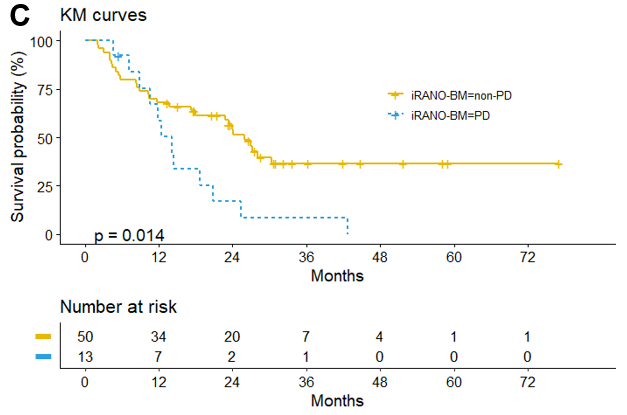

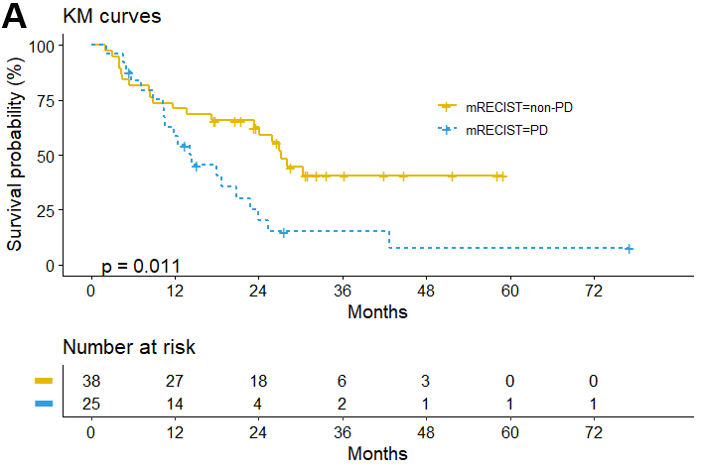


mRECIST, modified Response Evaluation Criteria in Solid Tumors; RANO-BM, Response Assessment in Neuro-Oncology Brain Metastases; iRANO-BM, immunotherapy Response Assessment in Neuro-Oncology adjusted for Brain Metastases
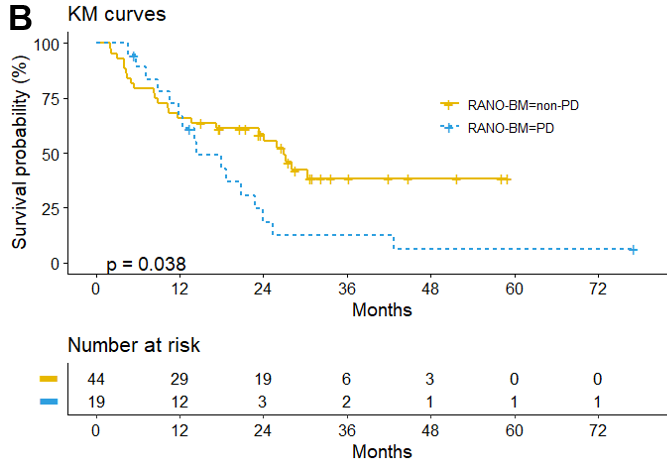
.

**
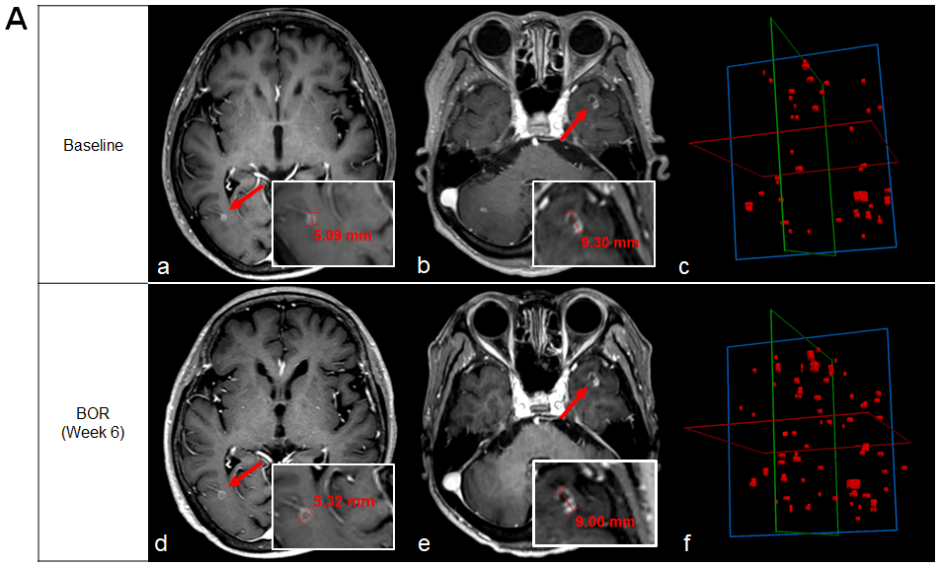
**

**Figure 4.** Representative Cases of 3D Response Utility in Predicting Overall Survival

**A.** Baseline scan (a, b) shows two target lesions of 5 and 9 mm. There were also other multiple lesions of less than 5 mm that are not shown in the image. On the MRI at best overall response (BOR) date (d, e), the previous target lesions show no significant change in size, and other multiple enhancing lesions measuring 1–2 mm have appeared (not shown in the image). Volumetric analysis of all segmented measurable and non-measurable enhancing lesions at baseline and the BOR date is shown in (c) and (f). On the baseline date (c), the total volume was 1459.1 mm^3^, and the total number of lesions was 35. On the BOR date (f), the total volume increased by 81.2% to 2643.4 mm^3^, and the measured number of lesions was 60. The patient died about 10 months after the baseline date.

**
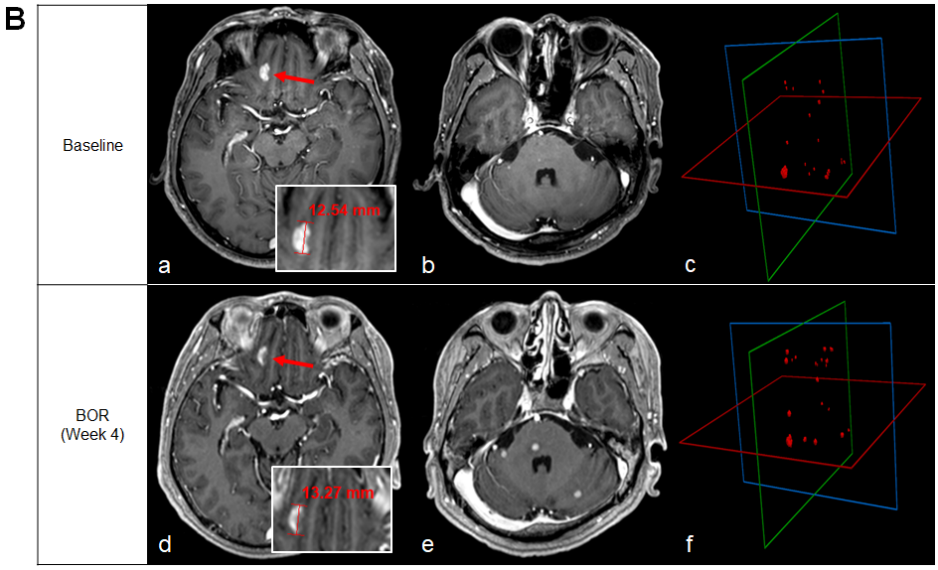
**

**B.** Baseline scan (a, b) shows a target lesion in the right frontal lobe with size of 12mm and other multiple lesions with size of less than 5mm. In the MRI at BOR date (d, e), previous target lesion and other multiple non-measurable enhancing lesions shows unequivocal size increase. Volumetric analysis of all segmented measurable and non-measurable enhancing lesions at baseline and the BOR date is shown in (c) and (f). On the baseline date (c), the total volume was 778.5mm^3^ and the lesion number 16. On the BOR date (f), the total volume increased by 70.8% to 1329.4mm^3^, and the measured number of lesions was 21. The patient died about 14 months after the baseline date.
