# Supplementary material for: Prognostic value of MRI volumetric parameters in non-small cell lung cancer patients after immune checkpoint inhibitor therapy: comparison with response assessment criteria
Source: Cancer Imaging. 2023 Oct 24;23:102. doi: 10.1186/s40644-023-00624-0 (PMC10594817; doi:10.1186/s40644-023-00624-0)
Supplement: Supplementary file 2 — Supplementary Material 2 [file 40644_2023_624_MOESM2_ESM.docx]

Dr. Raymond Huang, M.D., Ph.D.

Section Editor

Prof Rodney Hicks, M.D., Ph.D. and Prof Annick Van den Abbeele, M.D.Ph.D.

Editor-in-Chief

*Cancer Imaging*

October 5, 2023

Dear Editors,

Thank you for your recent correspondence regarding our manuscript, CAIG-D-23-0035. We enclose a revised version of our paper now entitled “Prognostic value of MRI volumetric parameters in non-small cell lung cancer patients after immune checkpoint inhibitor therapy: comparison with response assessment criteria”.

We have thoroughly edited and revised our manuscript to address the valuable comments and suggestions provided by the reviewers. In response to Reviewer 1, we have clarified the two co-primary purposes of our study. The first purpose was to measure and compare the responses of the different criteria for each patient. The second purpose was to investigate the association between the initial response measured by the subtraction method of sum of longest diameter (SLD)—indicated by the change in the diameters of target lesions between the BOR date and the baseline date—and overall survival. To avoid confusion, we have removed the term "response" when referring to the results of the subtraction method, and have used instead "change in SLDs" throughout the manuscript. Additionally, as suggested by Reviewer 2, we have updated the title and restructured the data presentation in the table.

We appreciate the valuable comments and helpful suggestions from the reviewers, which we believe have helped us to significantly improve the manuscript. We look forward to your reply.

Best regards,

Ji Eun Park, M.D., Ph.D.

Department of Radiology and Research Institute of Radiology, University of Ulsan College of Medicine, Asan Medical Center, 88 Olympic-ro 43-gil, Songpa-Gu, Seoul 05505, Korea

Phone: 82-2-3010-1505

E-mail address: [jieunp@gmail.com](mailto:jieunp@gmail.com)

**Response to Reviewers**

**Reviewer #1:**

1. 1) The authors' answer for the comment about the method determination of response (and using subtraction instead of ratio) was that baseline was often 0 with RANO-BM. However, with RANO-BM, the response is still not defined by a subtraction method when the baseline measurement is 0. Such patients would have SD as their best response (i.e., no PR or CR), and progression is identified by the appearance of any new measurable lesions or unequivocal progression of nontarget lesions. Therefore, it would still be inaccurate to define response using a subtraction method. For example, a difference between (5 mm and 9 mm) and (25 mm and 29 mm) is 4 mm. However, the first would qualify as PD (increase by almost 100%), and the second as SD (increase by <20%).

2) If the authors used the criteria suggested by RANO-BM, they can mention in the text that they determined response based on the published recommendations of RANO-BM, without mentioning the subtraction or ratio methods. This is what was actually used in Figure 4, where the authors commented on the percentage of increase rather than the absolute increase in size.

🡪 Thank you for this insightful comment. We agree with your opinion that it is inaccurate to define responses using a subtraction method. However, the subtraction method was not used to assess the response of each patient. The study had two purposes. The first purpose was to measure and compare responses using RECIST, RANO-BM, and iRANO adjusted for BM (iRANO-BM) for each patient. The second purpose was to investigate whether there is an association between the initial response measured by the subtraction method of SLD (sum of longest diameter)—indicated by the change in the diameter of target lesions between the BOR date and the baseline date—and overall survival. This was conducted irrespective of the results of each response assessment criterion.

Although the RANO-BM criteria includes an unequivocal increase in ‘non-target’ lesions for response assessment, this is essentially a qualitative evaluation. Given the limitation in measuring all non-target lesions, particularly those of sub-millimeter size, we focused solely on the changes in target lesions.

As the reviewer pointed out, the absolute difference in target lesion size changes from 5 mm to 9 mm and from 25 mm to 29 mm was 4 mm in both cases, yet they were classified differently under the response assessment. This discrepancy could be one of the reasons why we did not observe a significant association between the subtraction-method of SLDs and overall survival, which was the second purpose of our study. However, it is noteworthy that the change in volumetry demonstrated a significant association between the difference in target lesion diameter between the BOR date and baseline date and overall survival. We have clarified that the response assessment was assigned per each criterion in each patient and separated analysis was conducted for the subtraction method. We have deleted all mentions of “response” when referring to the subtraction method to avoid confusion and have used instead “change in SLDs” throughout the manuscript. We have added the study design to the Methods section as follows:

“*Study Design*

The study had two co-primary purposes. The first purpose was to measure and compare responses using different response criteria: RECIST, RANO-BM, and iRANO-BM for each patient. The second purpose was to investigate whether there is an association between the initial response measured by the subtraction method of the sum of longest diameter (SLD)—indicated by the change in the diameter of target lesions between the BOR date and the baseline date—and overall survival.*”*

In response to your second comment, we adopted a subtraction-based method and not a ratio-or percentage-based method ([*diameters at BOR* − *diameters at baseline*]/*diameter at baseline*) because in patients with BM, multiple small lesions are present at baseline and these are considered non-measurable under RANO-BM criteria, which yields a denominator of 0. For volumetry assessment, such as that shown in figure 4, both subtraction methods and percentage-based methods can be helpful. We have added this point in the Discussion section as follows:

“We adopted a subtraction-based method and not a ratio-based method ([*diameters at BOR* − *diameters at baseline*]/*diameter at baseline*) because in patients with BM, multiple small lesions are present at baseline and these are considered non-measurable under RANO-BM criteria, which yields a denominator of 0. For volumetric analysis, both the subtraction-based method and ratio (percentage)-based method are feasible.”

2. I apologize if I misunderstood the statement about the six patients who had PD by mRECIST and not RANO-BM. The authors mentioned that 5 of these 6 patients had SD and 1 had PR by RANO-BM, then added 1 who had PD by RANO-BM, making them 7 patients. Also, it is weird that 1 patient had PD by mRECIST (defined by progression of lesions that might be not measurable, and hence nontarget, by RANO-BM), and had PR by RANO-BM, when, by RANO-BM, PR requires stability of nontarget lesions. A progression of non-target lesions is also accounted for in RECIST. Can the authors comment on this?

🡪 We apologize for any confusion that our previous response may have caused. There appears to have been an error in the interpretation of your initial question, which led to incorrect calculations on our part.

Firstly, we appreciate you drawing our attention to this discrepancy. Despite the reduction in size of the target lesion, the appearance of a new lesion should indeed have been categorized as SD in accordance with RANO-BM criteria. We have corrected this information in the revised manuscript.

In response to your original query about the number of patients evaluated as having PD of non-target lesions per RANO-BM criteria, we wish to clarify that no patients were assessed as having PD based solely on the unequivocal progression of non-target lesions according to RANO-BM criteria. All patients also demonstrated an increase in the sizes of target lesions.

3-1. The primary objective of the study was to assess the correlation between volumetric response assessment and OS. However, it is not clear how BOR was determined. What criteria did the authors used in their volumetric assessment to determine BOR? Did they use any of the criteria used for 2D assessment (RECIST, RANO-BM, or iRANO-BM), or did they define BOR by the MRI with the least amount of disease compared to baseline? Did they use certain cutoff values or percentages to define a response?

🡪 We appreciate your query about the criteria used to determine BOR in our study. In our volumetric assessment, the BOR date was taken to be the time point of the smallest sum of the lesion volumes. This detail has been added to the Methods section of the revised manuscript as follows:

*“Quantitative Assessment: Changes in SLDs and Volumetric Measurements*

For the mRECIST, RANO-BM, and iRANO-BM criteria, the “diameter-based change in SLDs” were calculated as follows: (*SLD of the target lesions at BOR date*) − (*SLD of the target lesions at baseline date*). A similar calculation was applied for the volumetric measurement: (*total tumor volume at BOR date*) − (*total tumor volume at baseline date*). The BOR date of the volumetric assessment was taken to be the time point of the smallest sum of the lesion volumes.

3-2. Also, if the authors measured all the lesions in volumetrics, even the ones that did not qualify as target lesions in the 2D assessment with any of the criteria, this might have accounted for the difference in association with OS, as there are a lot of lesions not accounted for by the 2D assessment. Although this approach does not negate the benefit of volumetrics, it would make the case for advocating for assessing all the lesions (whether 2D or 3D), and not only measurable or certain measurable lesions, rather than volumetrics being better than 2D.

🡪 We appreciate this insightful comment and agree that the inclusion of all lesions in volumetric analysis could have influenced the results. Accordingly, we have added the following text to the Discussion section of the revised manuscript to clarify this point

“This discrepancy can be attributed to two primary factors. First, volumetric measurements may offer a more accurate representation of changes in tumor burden than 2D diameter changes. Second, volumetric assessments included all lesions, whereas 2D-based measurements were restricted to target lesions. In routine clinical practice, it is often impractical to measure all lesions, especially those with sub-millimeter diameters, and consistent tracking of these small lesions in follow-up imaging presents additional challenges. Given these considerations, volumetric analysis stands out as a valuable method for the more accurate and straightforward assessment of lesions.”

**Reviewer #2:**

Thank you for your thorough evaluation of the comments provided in the initial review. It appears that the main concerns were addressed appropriately.

1. The only potential update for clarity on this read is in Table 2. Although mentioned in the body of the manuscript, including (n=63) in the table title could help contextualize the results within the table. Additionally, it could read more cleanly if - at least for the ORR and disease control rate - the percentage were listed first then the N; e.g., 65% (41) for mRECIST disease control rate.

🡪 We appreciate these constructive comments aimed at enhancing the clarity of Table 2. To address your suggestions, we have included the sample size (n = 63) in the table's title and rearranged the order of presentation of the data so that the percentage values now precede the absolute counts.
